# Chloroform‐Assisted Rapid Growth of Vertical Graphene Array and Its Application in Thermal Interface Materials

**DOI:** 10.1002/advs.202200737

**Published:** 2022-03-24

**Authors:** Shichen Xu, Ting Cheng, Qingwei Yan, Chao Shen, Yue Yu, Cheng‐Te Lin, Feng Ding, Jin Zhang

**Affiliations:** ^1^ Center for Nanochemistry Beijing Science and Engineering Center for Nanocarbons Beijing National Laboratory for Molecular Sciences College of Chemistry and Molecular Engineering Peking University Beijing 100871 P. R. China; ^2^ Beijing Graphene Institute (BGI) Beijing 100095 P. R. China; ^3^ Key Laboratory of Marine Materials and Related Technologies Zhejiang Key Laboratory of Marine Materials and Protective Technologies Ningbo Institute of Materials Technology and Engineering (NIMTE) Chinese Academy of Sciences Ningbo 315201 P. R. China; ^4^ College of Chemistry and Chemical Engineering Ningxia University Yinchuan 750021 P. R. China; ^5^ School of Materials Science and Engineering Ulsan National Institute of Science and Technology Ulsan 44919 Korea; ^6^ School of Materials Science and Engineering Peking University Beijing 100871 P. R. China

**Keywords:** chloroform assisted rapid growth, electrical field, thermal interface materials, vertical graphenen arrays

## Abstract

With the continuous progress in electronic devices, thermal interface materials (TIMs) are urgently needed for the fabrication of integrated circuits with high reliability and performance. Graphene as a wonderful additive is often added into polymer to build composite TIMs. However, owing to the lack of a specific design of the graphene skeleton, thermal conductivity of graphene‐based composite TIMs is not significantly improved. Here a chloroform‐assisted method for rapid growth of vertical graphene (VG) arrays in electric field‐assisted plasma enhanced chemical vapor deposition (PECVD) system is reported. Under the optimum intensity and direction of electric field and by introducing the highly electronegative chlorine into the reactor, the record growth rate of 11.5 µm h^−1^ is achieved and VG with a height of 100 µm is successfully synthesized. The theoretical study for the first time reveals that the introduction of chlorine accelerates the decomposition of methanol and thus promotes the VG growth in PECVD. Finally, as an excellent filler framework in polymer matrix, VG arrays are used to construct a free‐standing composite TIM, which yields a high vertical thermal conductivity of 34.2 W m^−1^ K^−1^ at the graphene loading of 8.6 wt% and shows excellent cooling effect in interfacial thermal dissipation of light emitting diode.

## Graphical abstract

With a chloroform‐assisted method, a record growth rate of 11.5 µm h^−1^ was achieved and vertical graphene arrays with a height of 100 µm was successfully synthesized. Vertical graphene together with polydimethylsiloxane were used to construct a free‐standing thermal interface material, which yielded a high vertical thermal conductivity of 34.2 W m^−1^ K^−1^ and excellent cooling effect in light emitting diode.

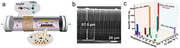

## 1 Introduction

With the rapid development of electronic devices toward high power density and miniaturization, thermal interface materials (TIMs), which function between a heating device and a heat‐sink, is getting more and more important along with the technology evolution.^[^ ^1^, ^2^, ^3^ ^]^ Graphene, yielding an outstanding in‐plane thermal conductivity, is widely used for thermally conductive materials.^[^ ^4^, ^5^, ^6^, ^7^ ^]^ However, graphene is always used as a thermal additive mixed with polymers to construct a composite thermal interface material (TIM).^[^ ^1^, ^8^, ^9^, ^10^ ^]^ Unfortunately, graphene framework will not significantly improve the performance of TIMs without special structural design.^[^ ^11^, ^12^ ^]^ Actually, to transfer intrinsic thermal property of nano graphene sheets into macroscopic graphene‐based TIMs, especially in vertical direction, the assembly strategy and structural design method of graphene framework play an important role.^[^ ^13^ ^]^ In principle, vertical graphene (VG) may significantly improve heat transfer capability.^[^ ^14^ ^]^ However, in top‐down method including rolling‐cutting method, directional‐freezing method, oriented hydrothermal reduction method and shrinking‐compressing method, VG is synthesized with graphene oxide or reduced graphene oxide dispersion assisted by polymer and solution, in where defects in graphene sheets and interfaces between graphene and polymer are harmful to the thermal properties of TIMs.^[^ ^15^, ^16^ ^]^ Instead, plasma enhanced chemical vapor deposition (PECVD), as a typical bottom‐up method, can directly grow VG structure on substrates retaining the intrinsic thermal properties in macro‐scale vertical materials.^[^ ^17^, ^18^ ^]^ Therefore, PECVD can be regarded as an effective method to prepare thin TIMs embedded with vertically aligned graphene framework at a film thickness in the range of several hundred micrometers.

As a well‐established industrially compatible thin‐film deposition technique, PECVD including radio frequency (RF), microwave (MW), and direct current (DC) plasma have been used to grow VG.^[^ ^19^, ^20^, ^21^ ^]^ MW and RF are often chosen as plasma sources to improve the growth rate of VG because of their high electron density and electron energy. However, VG grown by this method still faces the dilemma that the morphology of the grown graphene is difficult to control.^[^ ^22^, ^23^, ^24^ ^]^ DC PECVD and capacitively coupled PECVD are advantageous to the directional growth of VG due to their symmetrical electrode set‐up. Whereas, these methods are still stuck with limited growth rate.^[^ ^25^, ^26^ ^]^ Apparently, achieving rapid and directional growth of VG simultaneously using PECVD is still a long‐standing bottleneck.^[^ ^17^ ^]^

Herein, we developed a halogen‐regulated method for rapid growth of VG in an adjusted electric‐field‐assisted PECVD technique. Theoretical simulation was adopted to investigate the effect of electric field direction on growth rate. When the in‐built electric field is set to vertical upward, the grow rate of VG can be increased into 5 µm h^−1^. Furthermore, chloroform was used to accelerate growth rate of VG. When appropriate proportion of chloroform was introduced into methanol carbon source, the growth rate of VG could be increased to 11.5 µm h^−1^, resulting in a near 100‐µm VG array. Density functional theory (DFT) calculation results further proved that active species decomposed from chloroform consume the excessive gas‐phase H radical and remove excessive terminating H atom of a growing graphene edge for rapid growth of VG arrays. Finally, using the grown VG arrays, we prepared a VG composites‐based thermal interface material (TIM) by adding silicone. This TIM yield a high vertical thermal conductivity of 34.2 W m^−1^ K^−1^ at the graphene loading of 8.6 wt%, giving an ultrahigh thermal conductivity enhancement (TCE) per 1 wt% graphene over 2200%, superior to almost graphene composite‐based TIM. In addition, its better cooling effect than that of commercial thermal pads makes it has great potential in interfacial thermal dissipation of electronic products.

## 2 Results and Discussion

**Figure 1:**
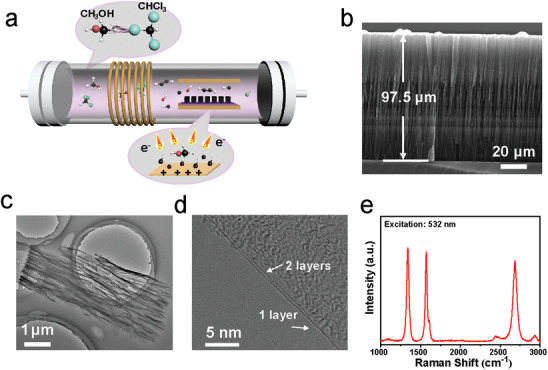
Chlorine‐assisted synthesis process and growth results of VG. a) Schematic illustrating the adjusted electric‐field‐assisted PECVD and growth procedure of VG in chlorine system. b) Side‐view SEM, c) low‐resolution TEM, d) high‐resolution TEM, and e) Raman spectrum of VG.

As is shown in **Figure** **1**a, induced coupled plasma generator combined with a CVD furnace constitutes our experiment set‐up. chloroform and methanol are directly introduced in our system for growth of VG. In addition, a built‐in electric field is also introduced into PECVD system inducing aligning force for vertical growth of VG arrays.^[^ ^27^ ^]^ As shown in Figure 1b, scanning electron microscope (SEM) image depicts that the graphene sheets completely perpendicular to the substrate and the height can reach nearly 100 µm. Aligned graphene sheets with bigger aspect ratio can be found in low‐resolution transmission electron microscope (TEM) image (Figure 1c and Figure S1, Supporting Information), suggesting that this method can always ensure the alignment of grapheme sheets even if its growth rate is significantly promoted by chloroform. The high‐resolution TEM images further exhibit few‐layer graphene sheets, even some of which are single layer and two layers (Figure 1d and Figure S2, Supporting Information). The Raman spectra shown in Figure 1e demonstrates that as‐grown VG exhibits high quality, whose intensity ratio of D peak to G peak is 1.04 and intensity ratio of 2D peak to G peak is 0.91.

### 2.1 Effect of Electric Field

**Figure 2:**
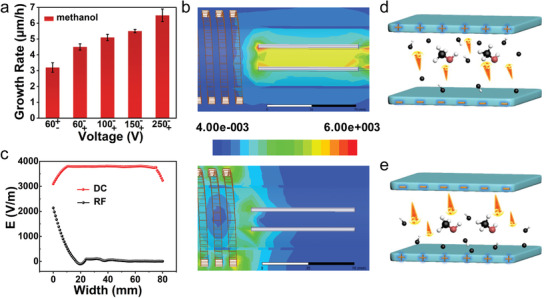
Effect of electric field on growth. a) Column chart showing the growth rate influenced by direction and intensity of electric field. b) Distribution of the electric field induced by DC electrode (left) and RF coil (right). c) Comparison of electric field intensity induced by DC electrode and RF coil. d,e) Plasma distribution and electron flow direction in opposite DC electric field.

The applied electric field induced the directional growth of graphene sheets and simultaneously regulated the growth rate of VG. As shown in **Figure** **2**a, the growth rate is 3.1 um h^−1^ at the voltage of $60\;V_{-}^{+}$ and increases to 4.5 um h^−1^ when the electric field direction is inversive ($60\;V_{+}^{-}$), suggesting that the direction of electric field affects the growth rate of VG. Besides, the growth rate gradually accelerated with the increased voltage intensity from 100 to 250 $V_{+}^{-}$ (Figure S3, Supporting Information).

In this work, the electric field was mainly generated by the RF coil and built‐in DC power system. To further explore the effect of electric field on the growth rate of VG, high‐frequency structure simulator (HFSS) has also been used to simulate the electric field distribution in the complicated plasma system. The vector diagram (Figure S4, Supporting Information) and distribution of the electric field induced by DC (Figure 2b, left) shows that the absolute intensity of DC electric field (*E*_DC_) is invariant even if the direction was reversed. While for the electric field induced by RF coil (*E*_RF_), its intensity and direction vary continuously with time Sine (Figure S5, Supporting Information). And the intensity of *E*_RF_ is only related to the distance between substrate and RF coil. It can be found in Figure 2b (right), electric field induced by coil on substrate almost disappear. Compared with the *E*_DC_ of 4000 V m^−1^, the *E*_RF_ around the substrate can be ignored, (Figure 2c) suggesting that *E*_DC_ play a dominant role in VG's growth by controlling the distribution of plasma in the growth system, especially active electrons. Figure 2d,e illustrates the movement direction of active electrons, moving from negative plate to positive plate under *E*_DC_. More high‐energy electrons mean a greater chance of collisions with carbon species. As illustrated in Figure 2d, when the upper electrode is positive, the electrons will move upward and there will be a large concentration of electrons, resulting in graphene growth on the upper plate. In contrast, as shown in Figure 2e, when the lower electrode is positive, a large number of electrons move toward the positive plate, resulting in a large number of carbon species near the substrate for rapid growth of VG.

### 2.2 The Effect of Chloroform

**Figure 3:**
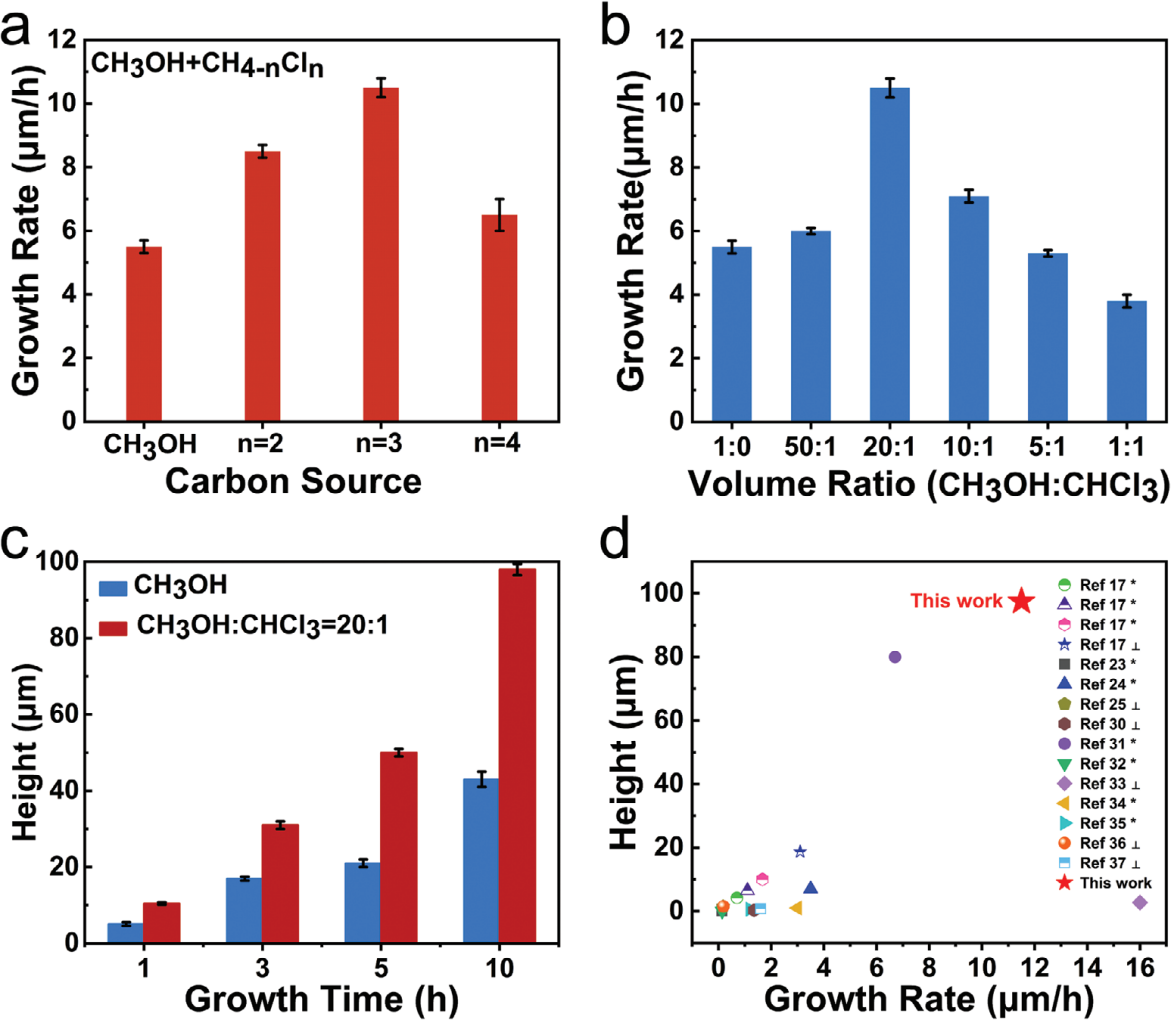
Rapid growth of VG assisted by chloroform. Effects of a) number of chlorine atomic and b) volume ratio of chloroform to methanol on VG growth. c) Histogram comparing the heights of VG arrays grown w and w/o chloroform versus growth time. d) Comparison of height, growth rate and morphology of VG in our work with those grown with traditional RF PECVD.

The rate of decomposition of alkanes is kinetically determined by the dehydrogenation reaction. The electronegative atoms are more likely to interact with hydrogen atoms and facilitate dehydrogenation reaction.^[^ ^28^, ^29^ ^]^ Based on this, considering the strong etching effect of fluorine plasma on silica, chlorine is regarded as the best choice for decomposition of methanol. **Figure** **3**a confirms that introduction of chlorine atoms can significantly accelerate growth of VG. Compared to dichloromethane, the growth rate of VG was increased dramatically using chloroform due to a bigger ratio of chlorine to carbon. However, the growth rate can't be prospectively increased when using tetrachloromethane due to the chemical reaction occurs between tetrachloromethane and methanol (Figure S6, Supporting Information). As can be found in Figure 3b, the growth rate of VG is gradually increased and reached a peak of 11.5 µm h^−1^ when the ratio of methanol to chloroform is 20:1. After that, the growth rate gradually slows down. (Figure S7, Supporting Information) This also indicates that moderate chlorine atoms facilitate the decomposition of methanol, while excess active carbon species inhibit the rapid growth of high‐quality VG (Figure S8, Supporting Information). Furthermore, VG at a height of 100 µm was finally prepared by increasing the growth time, which is much higher than those grown without chloroform at the same time (Figure 3c). Considering the growth rate, height, and morphology, the VG grown by electric‐filed‐assisted RF‐PECVD using mixed carbon source of methanol and chloroform has superior advantages over the previously reported VG grown via conventional RF‐PECVD method.^[^ ^17^, ^23^, ^24^, ^25^, ^30^, ^31^, ^32^, ^33^, ^34^, ^35^, ^36^, ^37^ ^]^ (Figure 3d and Table S1, Supporting Information).

**Figure 4:**
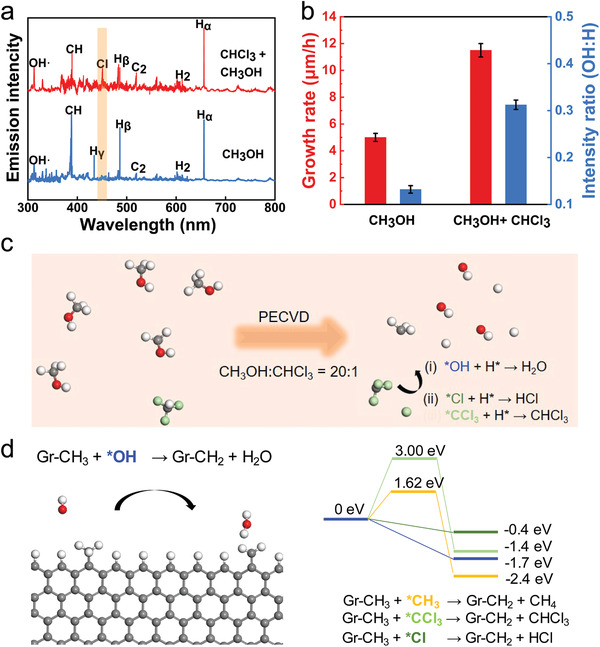
Mechanism of chloroform assisted rapid growth of VG. a) In situ OES spectra of the reaction systems. b)Comparison of growth rate and ration of *OH to H* between methanol and methanol‐chloroform system. c) Schematic diagram of gas‐phase species of graphene growth when the ratio of reactants CH_3_OH to CHCl_3_ is 20:1. The red, white, light green, and grey balls represent the O, H, Cl, and C atoms, respectively. d) Left panel: the relaxed geometry for the reaction of *OH radical. Right panel: the energy profile for the reaction of *OH (blue), *CH_3_ (orange), Cl* (dark green), and *CCl_3_ (light green) with a graphene zigzag edge to remove the additional edge H atom.

In order to reveal the growth mechanism, in situ optical emission spectrum (OES) was conducted directly to monitor the two different growth systems. **Figure** **4**a shows that signal peaks of OH, CH, C_2_, and series H can be found in both spectrums, but the signal peak of chlorine element at 452.6 nm can merely be detected in the chloroform assisted system, which is different from the non‐chloroform assisted system and is the main reason for improvement for growth rate.^[^ ^38^, ^39^ ^]^ In addition, in the chloroform system, the ratio of relative concentration of hydroxyl to hydrogen radical is 0.32, and the ratio of carbon species to hydrogen radical is 0.69, which is much higher than 0.12 and 0.53 in the non‐chloroform system, respectively. The more hydroxyl in the chloroform assisted system contributes to rapid growth of VG (Figure 4b).

### 2.3 Density Functional Theory (DFT) Calculations

Both X‐ray photoelectron spectroscopy (XPS) and TEM elemental analysis show that the graphene is chlorine‐free (Figures S9 and S10, Supporting Information), which implies that chlorine only plays an important role during growth process. To further explore the role of the chlorine in graphene growth, we performed density functional theory (DFT) calculations to reveal how chloroform changes the reaction kinetics during VG growth (see Method section). Figure 4c gives the schematic diagram of possible gas‐phase species during the graphene growth using the PECVD method when the ratio of reactants CH_3_OH to CHCl_3_ is 20:1. In the PECVD reactor, the plasma produces various active radicals such as CH*_x_* *(*x* = 0–3), H* and *OH in the gas phase. Moreover, *CCl_3_ and Cl* radicals are expected to exist considering the dehydrogenation of reactor CHCl_3_, which is also in good agreement with the OES signal of Cl* (Figure 4a) detected in experiments.

For the VG growth, all the reactions occur on the top edges of the graphene layers and thus the gas‐phase reaction is vital, which is similar with that of the vapor‐solid growth mechanism of graphene growth on the insulating substrates.^[^ ^40^ ^]^ As suggested in previous study,^[^ ^40^ ^]^ the threshold step of the VG is removing excessive hydrogen atoms from the graphene edge. To understand how the growth rate of VG was influenced by introducing suitable CHCl_3_, a zigzag graphene nanoribbon with hydrogen termination was constructed to present the growth front of graphene island for exploring the role of active radical *OH, *CH_3_, *Cl, and *CCl_3_ during the process of removing edge hydrogen atom. Figure 4d depicts the relaxed initial and final geometry of one *OH radical reacts with a graphene zigzag edge to remove the additional edge H atom by: Gr‐CH_3_ + *OH → Gr‐CH_2_ +H_2_O. It can be seen that this reaction is surprisingly barrierless (blue line of the right panel, Figure 4d), which suggests that more *OH radicals in the gas phase will be much beneficial for the growth of graphene. The reaction of active Cl* radical with the graphene edge H atom is also barrierless as shown in Figure 4d (green line of the right panel) and Figure S11a (Supporting Information). In comparison with *OH radical, the reaction barrier for possible *CCl_3_ and *CH_3_ radical is calculated to be about 3.00 eV (green line of the right panel in Figure 4d, relaxed geometry can be seen in Figure S11b, Supporting Information) and 1.62 eV (orange line of the right panel in Figure 4d), respectively. However, the whole growth is in the plasma‐enhanced environment, which will surely produce rich *H radical. Thus, the concentration of *OH will be much consumed by the H* radical in the gas‐phase (reaction (i) in Figure 4c and relaxed geometry in Figure S11c, Supporting Information). While with the assistance of reactant CHCl_3_ in our experiments, some chlorine species will be introduced, which could further react with the rich H* radical in the gas phase (reaction (ii) in Figure 4c and relaxed geometry in Figure S11d, Supporting Information), ensuring the concentration of *OH radical to some extent. The above calculations showed two key roles of CHCl_3_ in fast CVD growth of VG: first, in a plasma‐enhanced environment, the presence of CHCl_3_ will introduce some chlorine species, to consume the excessive gas‐phase H* radical, ensuring the concentration of *OH radical, which could assist the fast growth of VG; Second, active Cl* radical can also remove excessive terminating H atom of a growing graphene edge with no barrier. The two key roles ensure the fast growth of VG.

### 2.4 TIM Application of VG

**Figure 5:**
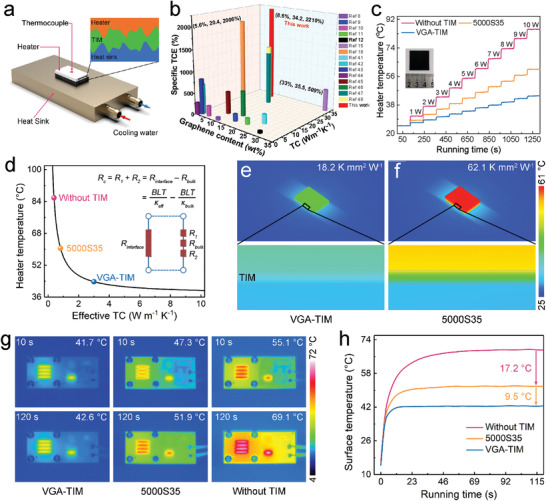
The performance of VGA‐TIM. a) Schematic illustrating the experimental configuration used for TIM performance evaluation. b) Histogram of thermal conductivity and specific TCE versus the graphene content. c) Surface temperature evolution of the heater as a function of applied powers, and the inset is the photograph of VGA‐TIM. d) Steady‐state temperature of heater versus effective thermal conductivity (TC) of different TIMs. e,f) Simulation temperature profiles showing the excellent cooling efficiency of VGA‐TIM. g,h) Operating temperature evolution of LED chips indicated by an IR camera.

In our work, the grown vertically aligned, covalently bonded vertical graphene arrays (VGA) were used as the filler skeleton for enhancing the through‐plane thermal conductivity of soft silicone matrix (polydimethylsiloxane (PDMS)) to form a high‐performance elastomer TIM (VGA‐TIM), whose real cooling efficiency was investigated by a home‐built measurement system developed for simulating the actual heat dissipation process of electronic devices, as shown in **Figure** **5**a. The VGA‐TIM as well as a commercial thermal pad (Bergquist 5000S35, USA, 5 W m^−1^ K^−1^) with a same bond line thickness (*BLT*) of 60 µm and a lateral size of 5 × 7 mm were placed between the heat sink and ceramic heater (5 × 7 × 1.2 mm), respectively, with a constant pressure of 60 psi. The circulating cooling water was continuously pumped into the heat sink for the full extraction of heat generated by the testing system, and the real‐time temperature variation was recorded by a thermocouple. As shown in Figure 5b and Table S2 (Supporting Information), our VGA‐TIM yielded a high vertical thermal conductivity of 34.2 W m^−1^ K^−1^ at the graphene loading of 8.6 wt%, achieving an ultrahigh specific thermal conductivity enhancement (TCE) over 2200% with per 1 wt% graphene content in polymer matrices, which is better than most graphene composite‐based TIM.^[^ ^8^, ^9^, ^10^, ^11^, ^12^, ^15^, ^18^, ^41^, ^42^, ^43^, ^44^, ^45^, ^46^, ^47^, ^48^ ^]^ Figure 5c presents the steady‐state surface temperatures of ceramic heater in different TIM cases as a function of applied powers. Observably, the VGA‐TIM (seen the inset of Figure 5b) always exhibited a superior capacity in heat dissipation than the commercial counterpart reflected by the significant temperature drops, which is finally reached up to 16.8 °C at a used power of 10 W. Note that these data also can be transformed into a linear form (Figure S12, Supporting Information), thus we can obtain the equivalent heat transfer coefficients (equal to the reciprocal of slope) based on the linear variation between heater temperatures and applied power densities, which are 1.53 (VGA‐TIM), 0.81 (5000S35), and 0.47 W cm^−2^ °C^−1^ (without TIM), respectively, demonstrating that the cooling efficiency of VGA‐TIM is ≈1.89 times higher than that of state‐of‐the‐art thermal pad.

A commercial computational fluid dynamics software (ANSYS Icepak) was employed to in‐depth investigate the effective thermal conductivities (*κ*_eff_) and thermal contact resistances (*R*_c_) of two samples, with an applied power of 10 W. The *κ*_eff_ of different cases can be obtained according to the steady‐state heater temperatures shown in Figure 5d. As a result, the value of VGA‐TIM is 3 W m^−1^ K^−1^, which is nearly 3.7 times higher than that of 5000S35 (0.81 W m^−1^ K^−1^). Therefore, we can calculate the *R*_c_ of the two samples by the following equation:

(1) $$R_{c}=\mathrm{BLT}/\kappa_{\mathrm{eff}}-\mathrm{BLT}/\kappa_{\mathrm{bulk}}$$

where the *κ*_bulk_ is the through‐plane thermal conductivities of VGA‐TIM/5000S35. As a result, the *R*_c_ value of VGA‐TIM is determined to 18.2 K mm^2^ W^−1^, showing a significant decrease of 70.7% compared with that of 5000S35 thermal pad (62.1 K mm^2^ W^−1^) under the same testing conditions (The detailed parameters used in the calculations were listed in Table S3, Supporting Information). Figure 5e,f are the comparative simulation temperature profiles of the cases used VGA‐TIM and 5000S35 as the TIMs, respectively, further confirming the superior heat dissipation capacity of our sample. Such excellent TIM performance of VGA‐TIM is attributed to not only higher through‐plane thermal conductivity, but also lower thermal contact resistance (29.3%) due to low compressive modulus of VGA‐TIM originated from very low filler content of 8.6 wt% (The TGA analysis can be found in Figure S13, Supporting Information), in which the latter can maintain the great compressive property of soft PDMS.

The VGA‐TIM was then used in the temperature reduction of a high‐power light emitting diode (LED, 10 W) to evaluate its heat transfer capacity in real operating conditions. In a typical experiment process, the LED lamp was fixed on the aluminum heat sink by four fastening screws, and the VGA‐TIM as well as 5000S35 with the same *BLT* and lateral size were used to fill the contact interface, respectively. The temperature evolution of LED was recorded by a calibrated infrared camera, and the corresponding results were displayed in Figure 5g,h. After the illumination of LED lamps, compared with other counterparts, the case used our sample as TIM not only rapidly achieves a steady state, but also has a lower saturation temperature (42.6 °C), which is much smaller than these cases without TIM (69.1 °C) and using 5000S35 (51.9 °C). Note that the reliability of LED depends exponentially on the operating temperature, in which every 10 °C temperature drop can result in 50% increase of lifetime. Accordingly, based on achieving a superior heat transfer capacity, our finding is a promising candidate to replace the state‐of‐the‐art thermal pad for cooling the high‐power LED lamps and electronic devices.

## 3 Conclusion

In conclusion, we demonstrated a halogen‐regulated method for rapid growth of VG in an adjusted electric‐field‐assisted PECVD system. When appropriate proportion of chloroform was introduced into methanol carbon source, the maximum growth rate of VG could be increased to 11.5 µm h^−1^. Later, 100‐µm VG arrays was achieved within 10 h. This study provides new insights into plasma and gas‐phase reaction engineering to obtain aligned graphene sheets and paves the way for the rapid growth of VG with high quality. More importantly, using the designed vertical graphene structure, we fabricate a VG based composite for TIM application, which exhibited excellent thermal properties and outstanding cooling effect, showing huge potential in interfacial heat dissipation of electronic devices.

## 4 Experimental Section

### Growth Method of VG

A conventional inductively coupled PECVD with a frequency of 13.56 MHz was adopted as the equipment for growth of vertical graphene (VG). Then a built‐in electric field was introduced into the system for alignment of graphene sheets. The electric field was provided by two electrodes connected to a direct current power supply. Voltage can be adjusted from 1 to 250 V. During the growth process, silicon wafers were placed in the heating zone of a tube‐type PECVD. After the growth temperature was up to 650 °C, the mixture carbon sources of methanol and chloroform were introduced into the system. Plasma was then generated with a radio frequency source power of 250 W. Following an electric field was set as a specific value, oriented VG arrays can be prepared.

### Calculation Details

The geometry relaxation and energy barrier calculations were performed under the framework of density functional theory (DFT) as implemented in Vienna ab‐initio Simulation Package (VASP). For the treatment of exchange‐correlation potentials, the Perdrew–Burke–Ernzerhof (PBE) functional within the generalized gradient approximation (GGA) was adopted. A zigzag graphene nanoribbon with the unit cell of 14.75 × 30.00 Å was constructed. A kinetic energy cutoff of 400 eV was used, and give a threshold convergence of 10–4 eV and 0.01 eV Å^−1^ for the energies and forces of the system, respectively. To fully include the effects of radicals and the graphene edge, spin‐polarization in the calculations was used. To give the reaction energy barrier, the climbing image nudged elastic band (CI‐NEB) approach was applied.

### General Characterization

The morphology and detailed structure of VG was investigated by SEM (FEI Quattro S, acceleration voltage 5–10 kV, TEM (FEI Tecnai F20; acceleration voltage 200 kV); Raman spectroscopy (Horiba, LabRAM HR 800, 532 nm laser wavelength), XPS (Kratos Analytical Axis‐Ultra spectrometer with Al K*α* X‐ray source). The thermal diffusivities (*α*) of the sample were measured using LFA 467 MicroFlash system (NETZSCH, Germany). The specific heat capacity of the sample evaluated by using a DSC (PYRIS Diamond, PerkinElmer, USA). The infrared (IR) photos were captured by using an infrared camera (Fluke, Ti400, USA).

### Statistical Analysis

Microsoft Excel and OriginPro (version 2019, Originlab Corp.) were used for the statistical analysis of the data presented in this work. Calculation method was detailed descripted in *Calculation Details*. All growth data in Figures 3a–c and 4b were presented as mean ± SD (standard deviation) and the sample size to be tested is listed in Table S4 (Supporting Information).

## Conflict of Interest

The authors declare no conflict of interest.

## Supporting information

Supporting Information

## Data Availability

The data that support the findings of this study are available in the supplementary material of this article.
