# Supplementary material for: Chloroform‐Assisted Rapid Growth of Vertical Graphene Array and Its Application in Thermal Interface Materials
Source: Adv Sci (Weinh). 2022 Mar 24;9(15):2200737. doi: 10.1002/advs.202200737 (PMC9130900; doi:10.1002/advs.202200737)
Supplement: Supplementary file 1 — Supporting Information [file ADVS-9-2200737-s001.pdf]

ADVANCED SCIENCE
Open Access

Supporting Information

for *Adv. Sci.*, DOI 10.1002/advs.202200737

Chloroform-Assisted Rapid Growth of Vertical Graphene Array and Its Application in Thermal Interface Materials

Shichen Xu, Ting Cheng, Qingwei Yan, Chao Shen, Yue Yu, Cheng-Te Lin, Feng Ding* and Jin Zhang**

Supporting Information

Chloroform Assisted Rapid Growth of Vertical Graphene Array and Its Application in Thermal Interface Materials

Shichen Xu,[1, 2, #] *Ting Cheng,*[1, #] *Qingwei Yan,*[4,#] *Chao Shen,*[2,6] *Yue Yu,*[1] *Cheng-Te Lin,*[4,*] *Feng Ding,*[3,*] *Jin Zhang*[1,2,5,*]

S. C. Xu, Dr. T. Cheng, Y. Yu, Prof. J. Zhang
[1]Center for Nanochemistry
Beijing Science and Engineering Center for Nanocarbons
Beijing National Laboratory for Molecular Sciences
College of Chemistry and Molecular Engineering
Peking University
Beijing 100871, P. R. China
E-mail: jinzhang@pku.edu.cn

S. C. Xu, C. Shen, Prof. J. Zhang
[2]Beijing Graphene Institute (BGI), Beijing, 100095, P. R. China

Prof. F. Ding
[3]School of Materials Science and Engineering
Ulsan National Institute of Science and Technology
Ulsan 44919, Korea
E-mail: f.ding@unist.ac.kr

Dr. Q. W. Yan, Prof. C. T. Lin
[4]Key Laboratory of Marine Materials and Related Technologies
Zhejiang Key Laboratory of Marine Materials and Protective Technologies
Ningbo Institute of Materials Technology and Engineering (NIMTE)
Chinese Academy of Sciences
Ningbo 315201, P. R. China
E-mail: linzhengde@nimte.ac.cn

Prof. J. Zhang
[5]School of Materials Science and Engineering
Peking University
Beijing 100871, P. R. China

C. Shen
[6]College of Chemistry and Chemical Engineering
Ningxia University
Yinchuan 750021, P. R. China

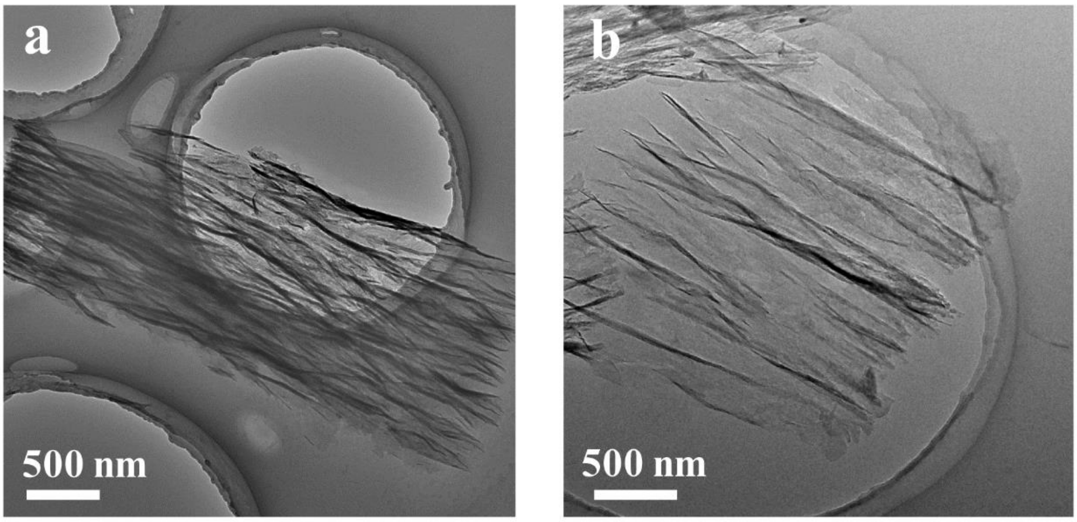

Figure S1. Comparison of VG grown w/o chloroform. (a) TEM images of VG grown with mixture of methanol and chloroform. (b) TEM images of VG grown with methanol. Graphene sheets grown with mixture of methanol and chloroform shows bigger aspect ratio.

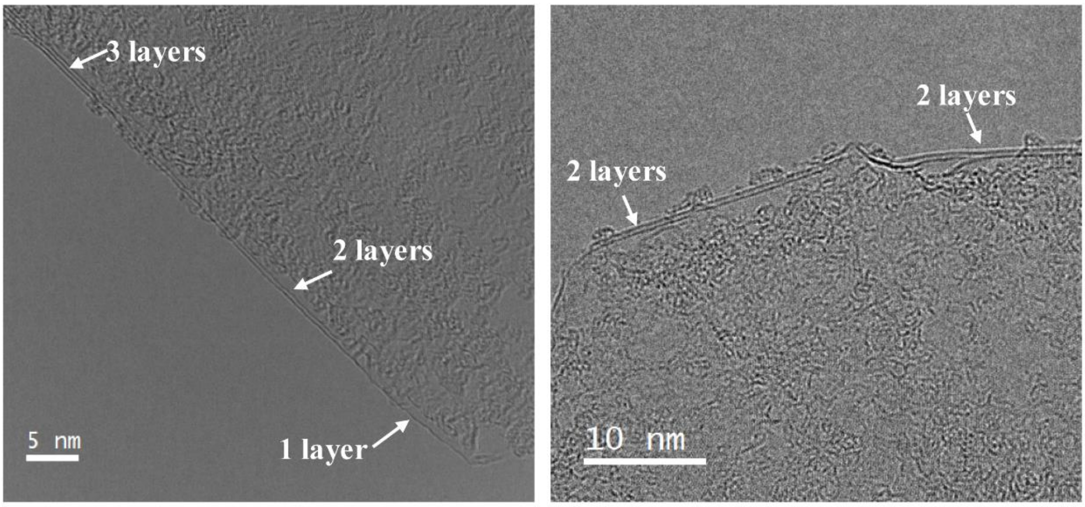

Figure S2. High-resolution TEM images of few-layer graphene sheets.

To pursuit vertical growth of graphene, we introduced a built-in electric field into the traditional RF-PECVD system by two electrodes connecting to the external DC power supply. The growth results show that both the direction and the intensity of electric field affect the growth rate of VG. In order to explore the influence of electric field distribution, we use high-frequency structure simulator to simulate electric field distribution in the system.

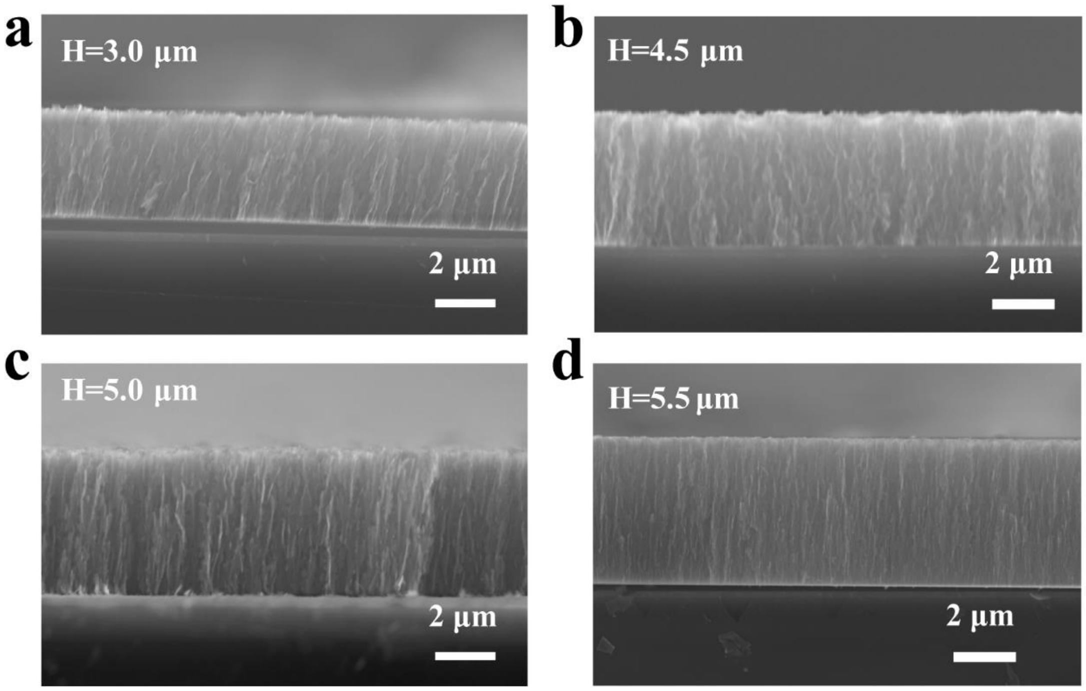

Figure S3. Sectional SEM images of VG grown assisted by electric field. (a) The bottom electrode is negative, the voltage is 60 V. (b-d) The bottom electrode is positive. The DC voltages applied to the electrodes are 60 V, 100 V and 150 V, respectively

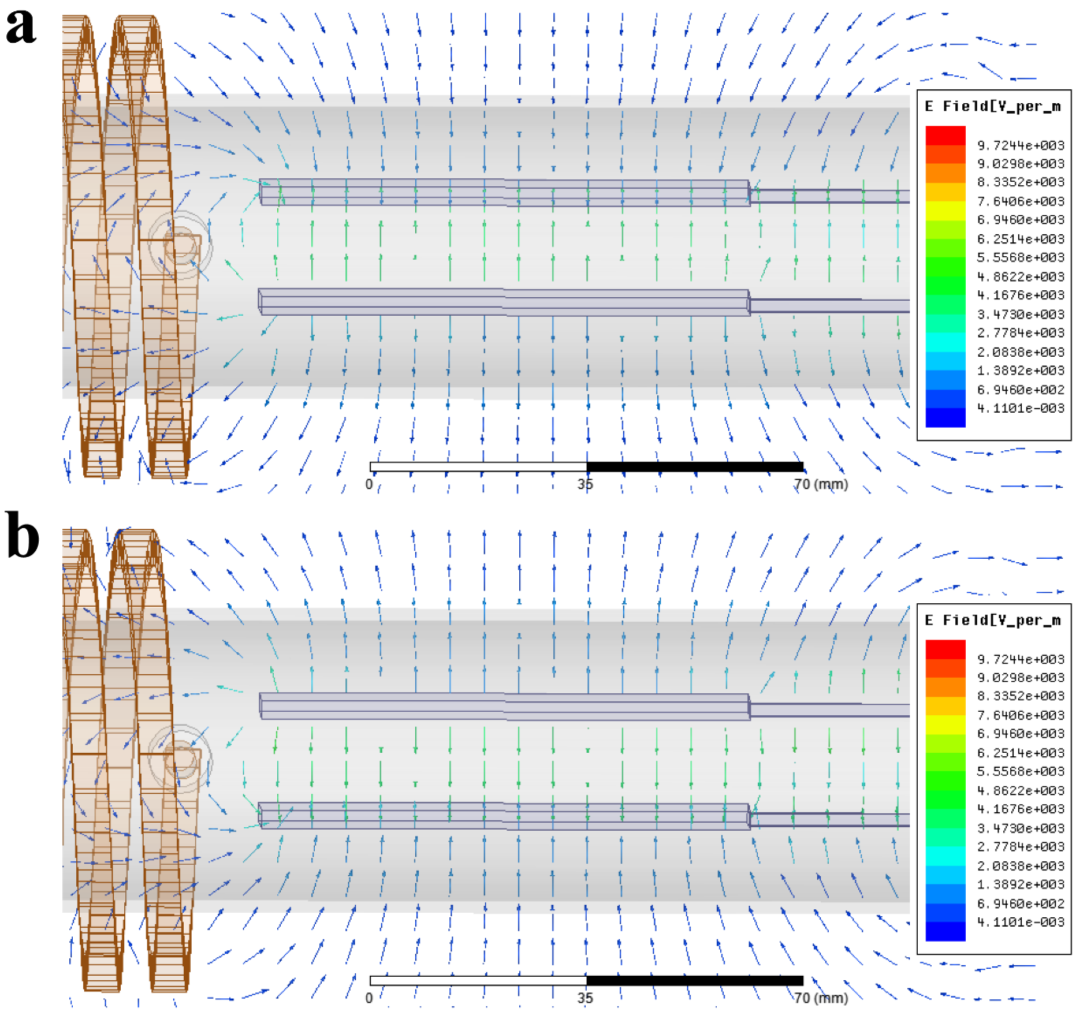

Figure S4. Vector diagrams of electric field distribution induced by two electrodes applied by DC voltages. (a) The bottom electrode is negative. (b) The bottom electrode is positive.

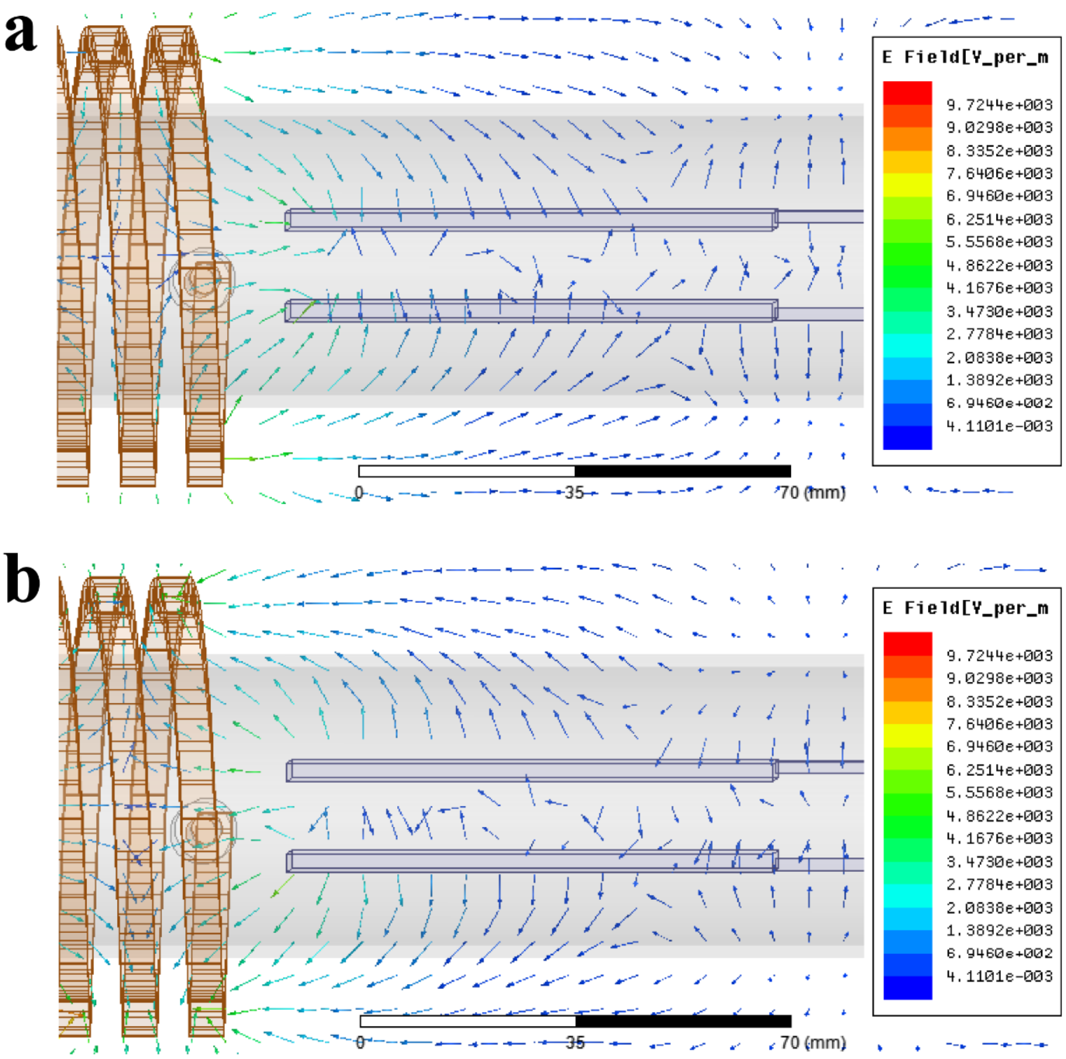

Figure S5. Vector diagrams of electric field distribution induced by RF coil. $E=E_0+\sin(\theta+\varphi)$ (a) $\varphi=0$, (b) $\varphi=180$.

Carbon sources are always the key parameter for carbon material growth. In this part, we mainly discuss the effects of chloroform on growth, including the concentration and volume ratio of chloroform to methanol on VG growth. Finally, compared with VG grown by tradition RF-PECVD method Considering the growth rate, height, and morphology, the VG grown by electric-filed-assisted RF-PECVD using mixed carbon source of methanol and chloroform has absolute advantages in growth rate, height, and morphology.

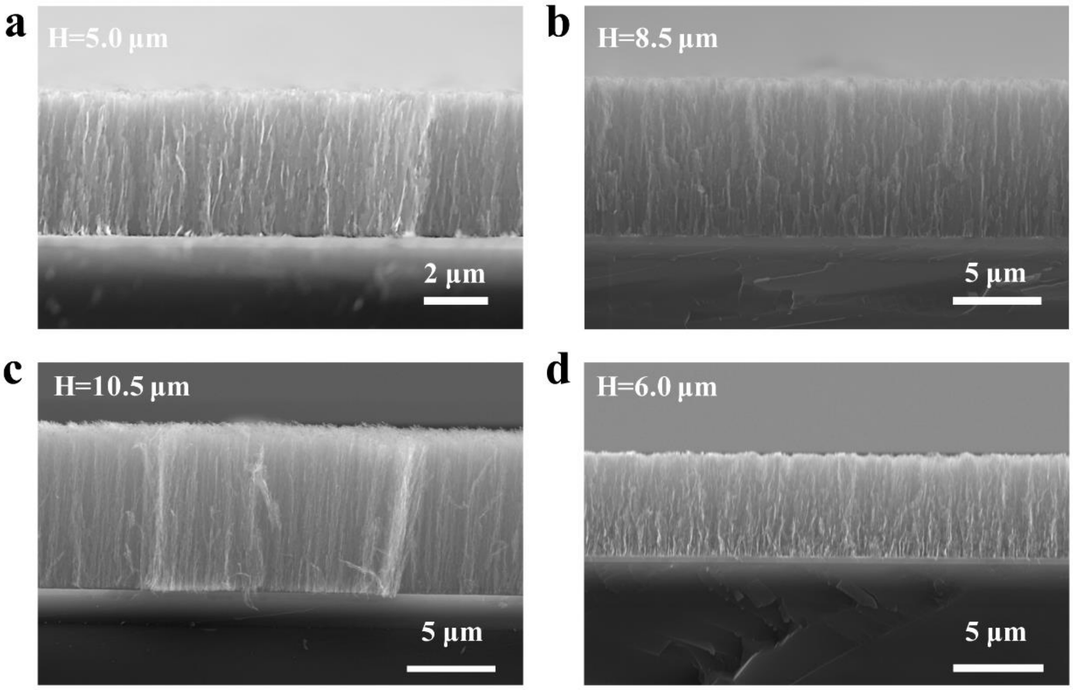

Figure S6. Sectional SEM images of VG grown with different carbon sources. (a) Methanol. (b)Methanol and dichloromethane. (c)Methanol and chloroform. (d)Methanol and tetrachloromethane.

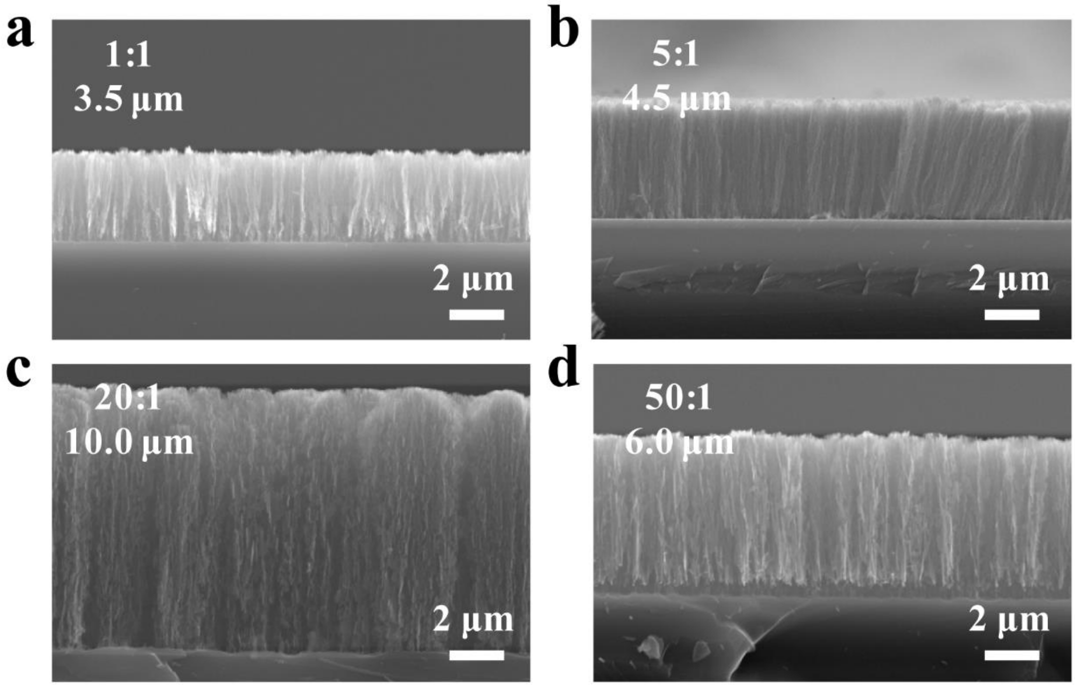

Figure S7. Sectional SEM images of VG grown with different chloroform concentration. Ratio of methanol to chloroform is (a) 1:1, (b) 5:1, (c) 20:1, (d) 50:1, respectively.

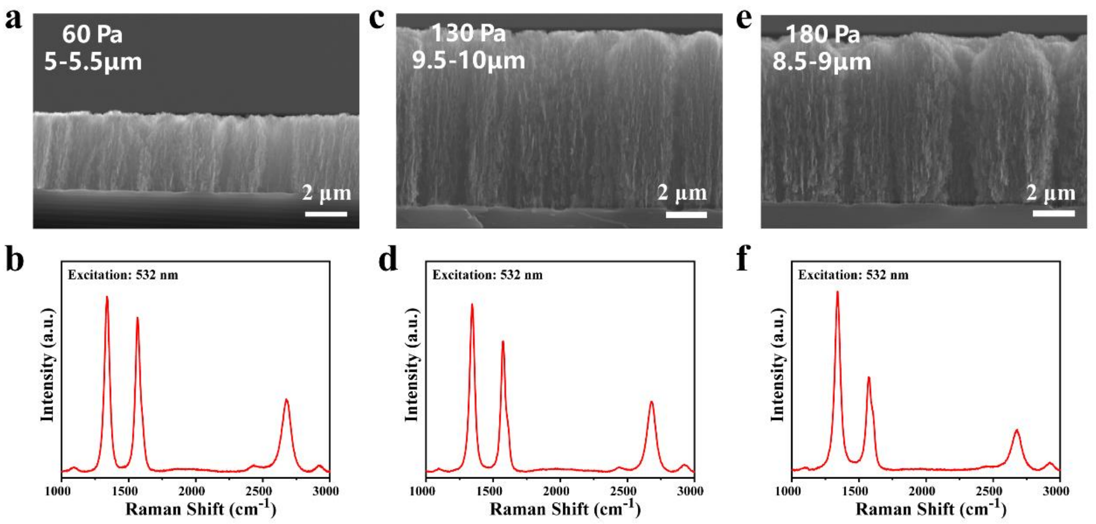

Figure S8. Sectional SEM images and Raman spectra of VG grown with different carbon concentration. The total pressures of carbon sources are (a, b) 60 Pa, (c, d) 130 Pa, and (e, f) 180 Pa, respectively. The growth result means that moderate chlorine atoms facilitate the decomposition of methanol, while excess active carbon species inhibit the rapid growth of high-quality VG.

Table 1. VG grown with RF-PECVD.

Plasma power	Carbon source	Growth time(h)	Height(μm)	Growth rate(μm/h)	vertical/ random	Ref.
280W	C_2H_2	0.2	0.27	1.35	vertical	1
500W	C_2H_2	12	80	6.7	random	2
280W	C_2H_2	2	7	3.5	random	3
300W	C_2H_5OH	0.5	0.07	0.14	random	4
1000W	C_2H_2	1/6	2.72	16	vertical	5
900W	CH_4	1/3	1	3	random	6
400W	CH_4	0.5	0.6	1.2	random	7
300W	CH_4	1	0.13	0.13	random	8
100W+400W	C_2F_6	8	1.4	0.175	vertical	9
100W+400W	CH_4\CFx	8	1.5	0.1875	vertical	10
250W+300W	C_2F_6	0.5	0.8	1.6	vertical	11
250W	methane	6	4.2	0.7	random	12
250W	ethanol	6	6.5	1.1	random	12
250W	methanol	6	10	1.67	random	12
250W	methanol	6	18.7	3.1	vertical	12
250W	methanol chloroform	9	97.5	11.5	vertical	this work

Given the introduction of heteroatoms into the system, it is critical to determine whether the graphene sheet is doped with heteroatoms. Both X-ray photoelectron spectroscopy and selected area electron diffraction show that there is no chlorine was doped into the graphene, even though the graphene was grown with chloroform.

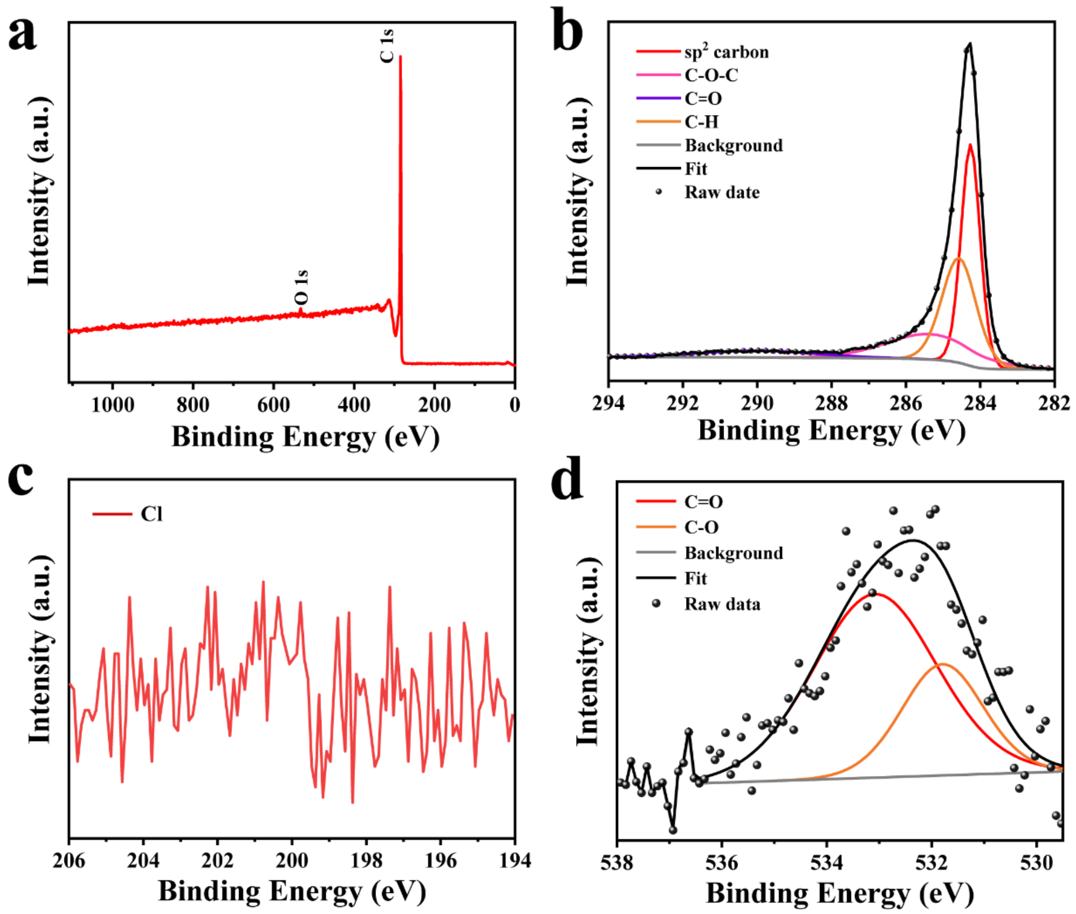

Figure S9. X-ray photoelectron spectroscopy (XPS) of the VG grown with chloroform and methanol. (a) XPS spectra of VG arrays shows that no impurity atoms are introduced into the VG arrays. (b) C1s spectrum. The C 1s spectrum contains a predominant sp2 carbon peak (284.6 eV), a C-H peak (285.2 eV), as well as a broad C=O peak and C-O peak. (c) Cl1s spectrum. No chlorine signal except noise signal. (d) O1s spectrum.

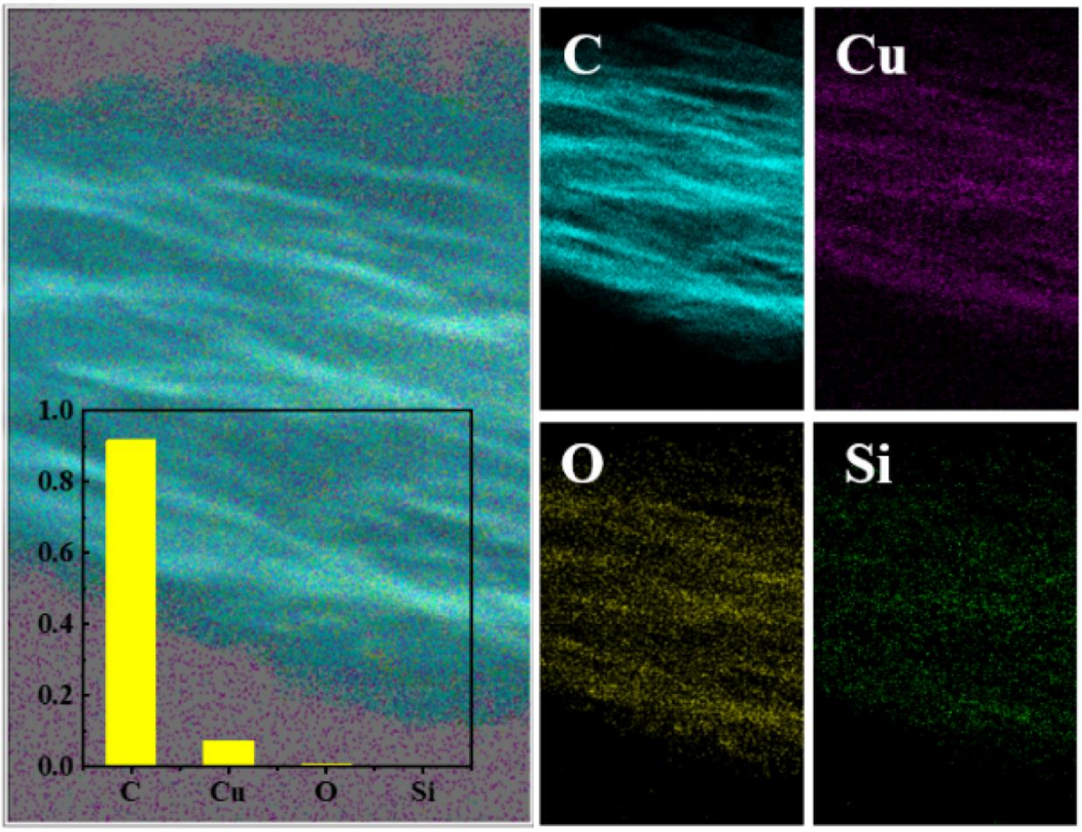

Figure S10. High-resolution TEM image and SAED image of VG arrays. The right pictures are EDS elemental mapping images of C, Cu, O, and Si in the VG arrays. No chlorine was detected.

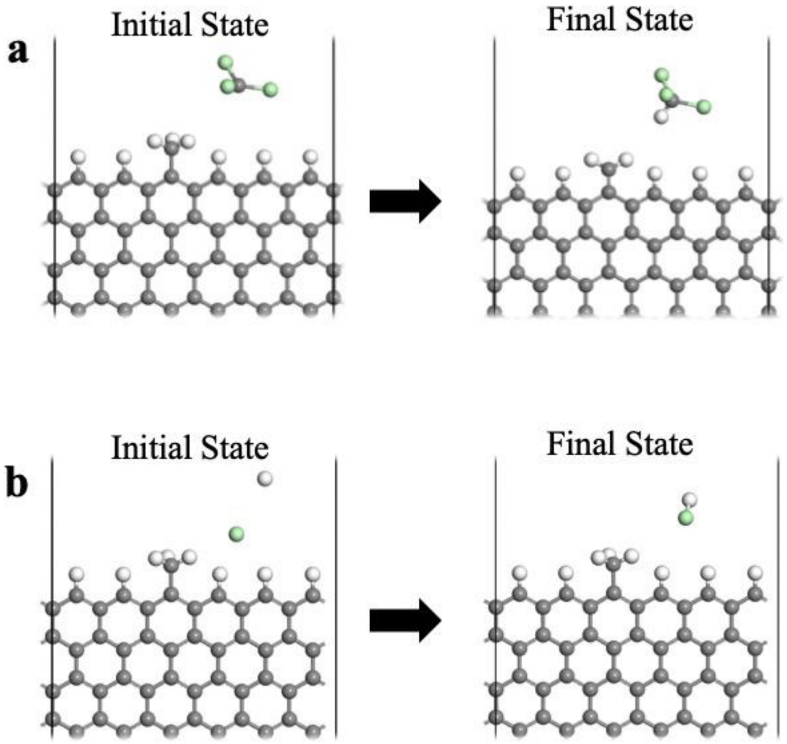

Figure S11. The relaxed geometry for the reaction of *CCl_3 and *Cl radical. (a) *CCl_3 radical reacts with the graphene edge. (b) *Cl radical reacts with H* in the gas phase.

Table 2. Thermal conductivity of graphene composite-based TIM.

Plomer resin	TC of plomer	TC of composite	Content of graphene	Specific TCE	Ref.
Rubber	0.2	2.9	27.8	49	13
Epoxy	0.2	1.53	10	67	14
Epoxy	0.2	5.8	20	140	15
Epoxy	0.2	5.1	15	163	16
PVDF	0.19	10.19	24	219	17
PDMS	0.175	2.19	4.82	239	18
Epoxy	0.21	35.5	33	509	19
Epoxy	0.23	2.69	1.55	690	20
Epoxy	0.16	2.13	1.5	821	21
PDMS	0.19	0.56	0.7	278	22
Epoxy	0.2	8.8	8.3	518	23
Rubber	0.13	10.64	13.9	582	24
PDMS	0.19	28.12	12	1225	25
PDMS	0.18	28.77	11.62	1367	26
PDMS	0.18	20.4	5.6	2006	27
PDMS	0.18	34.2	8.6	2210	This work

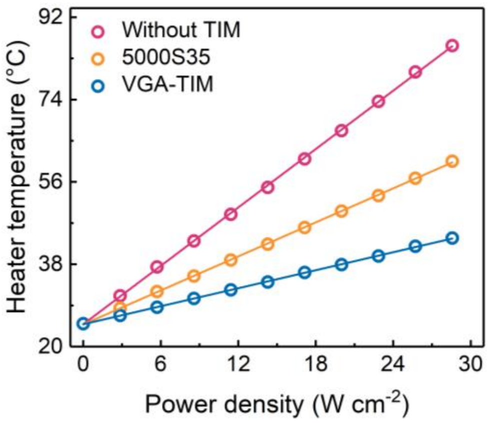

Figure S12. Surface temperature evolution of the heater as a function of applied power density.

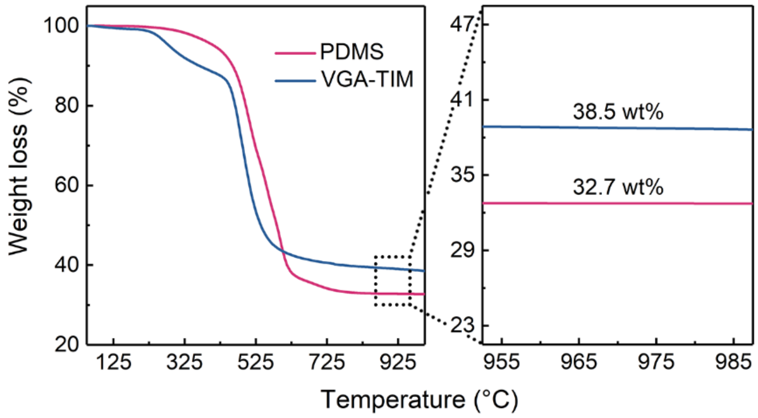

Figure S13. TGA curves of different samples. Considering that the residual mass of PDMS is 32.7% and the residual mass of the VGA/PDMS is 38.5%, the content of VG in the VGA /PDMS composite is calculated to be 8.6 wt%.[27]

Note that the VGA /PDMS composite is composed by that of the PDMS and VGA, namely:

$$m_{\mathrm{PDMS}} + m_{\mathrm{VGA}} = m_{\mathrm{VGA/PDMS}} \quad (1)$$

$m_{\mathrm{VGA/PDMS}}$ is the mass of the VGA/PDMS, and the m_{PDMS} as well as the m_{VGA} are that of the PDMS and VGA in the composite. The residual fraction of the VGA/PDMS composite (32.7 wt%) includes the VGA and the residual PDMS, which can be written as the following equation:

$$0.327m_{\mathrm{PDMS}} + m_{\mathrm{VGA}} = 0.385m_{\mathrm{VGA/PDMS}} \quad (2)$$

Thus, combining the equation 1 and 2, the content of VGA in the VGA/PDMS composite is calculated to be 8.6 wt%.

Table S3. The detailed parameters for calculations and the corresponding results.

Sample	*BLT* (μm)	κ_{bulk} (W m^{-1} K^{-1})	R_{bulk} (K mm^2 W^{-1})	κ_{eff} (W m^{-1} K^{-1})	$R_{interface}$ (K mm^2 W^{-1})	R_c (K mm^2 W^{-1})
VGA-TIM	60	34.2	1.8	3.0	20.0	18.2
5000S35	60	5.0	12	0.81	74.1	62.1

Table S4. The all detailed statistical analysis of this work.

Figure in manuscript	X axis	Sample size	Mean ± SD
Figure 3a	CH_3OH	5	5.5±0.2 μm
Figure 3a	n=2	5	8.4±0.2 μm
Figure 3a	n=3	5	10.5±0.2 μm
Figure 3a	n=4	5	6.6±0.2 μm
Figure 3b	1:0	5	5.5±0.2 μm
Figure 3b	50:1	5	6.0±0.1 μm
Figure 3b	20:1	10	10.5±0.3 μm
Figure 3b	10:1	5	7.1±0.2 μm
Figure 3b	5:1	5	5.3±0.1 μm
Figure 3b	1:1	5	3.8±0.2 μm
Figure 3c	1 (red)	15	10.5±0.3 μm
Figure 3c	3 (red)	5	31±1 μm
Figure 3c	5 (red)	5	50±1 μm
Figure 3c	10 (red)	5	98±1.5μm
Figure 3c	1 (blue)	5	5.1±0.5 μm
Figure 3c	1 (blue)	5	17±0.5 μm
Figure 3c	1 (blue)	5	21±1 μm
Figure 3c	1 (blue)	5	43±2 μm
Figure 4b	CH3OH (blue)	3	0.1321±0.0081
Figure 4b	CH3OH+ CHCl3 (blue)	3	0.3125±0.0103

REFERENCES

[1] C. Yang, H. Bi, D. Wan, F. Huang, X. Xie, M. Jiang, *J. Mater. Chem. A* **2013**, *1*, 770.

[2] Y. Ma, W. Jiang, J. Han, Z. Tong, M. Wang, J. Suhr, X. Chen, L. Xiao, S. Jia, H. Chae, *ACS Appl. Mater. Interfaces* **2019**, *11*, 10237.

[3] M. Hiramatsu, K. Shiji, H. Amano, M. Hori, *Appl. Phys. Lett.* **2004**, *84*, 4708.

[4] S. Xu, S. Wang, Z. Chen, Y. Sun, Z. Gao, H. Zhang, J. Zhang, *Adv. Funct. Mater.* **2020**, *30*, 2003302.

[5] Y. Ma, H. Jang, S. J. Kim, C. Pang, H. Chae, *Nanoscale Res. Lett.* **2015**, *10*, 1019.

[6] J. Han, Y. Ma, M. Wang, L. Li, Z. Tong, L. Xiao, S. Jia, X. Chen, *ACS Appl. Mater. Interfaces* **2021**, *13,* 12400.

[7] J. Shan, L. Cui, F. Zhou, R. Wang, K. Cui, Y. Zhang, Z. Liu, *ACS Appl. Mater. Interfaces* **2020**, *12*, 11972.

[8] M. Y. Zhu, R. A. Outlaw, M. Bagge-Hansen, H. J. Chen, D. M. Manos, *Carbon* **2011**, *49*, 2526.

[9] J. Zhao, M. Shaygan, J. Eckert, M. Meyyappan, M. H. Rummeli, *Nano. Lett.* **2014**, *14*, 3064.

[10] X. Song, J. Liu, L. Yu, J. Yang, L. Fang, H. Shi, C. Du, D. Wei, *Mater. Lett.* **2014**, *137*, 25.

[11] K. Shiji, M. Hiramatsu, A. Enomoto, M. Nakamura, H. Amano, M. Hori, *Diam. Relat. Mater.* **2005**, *14*, 831.

[12] S. Kondo, M. Hori, K. Yamakawa, S. Den, H. Kano, M. Hiramatsu, *J. Vac. Sci. Technol. B* **2008**, *26*, 1294.

[13] Y. Li, F. Xu, Z. Lin, X. Sun, Q. Peng, Y. Yuan, S. Wang, Z. Yang, X. He, Y. Li, *Nanoscale* **2017**, *9*, 14476.

[14] S. H. Song, K. H. Park, B. H. Kim, Y. W. Choi, G. H. Jun, D. J. Lee, B. S. Kong, K. W. Paik, S. Jeon, *Adv. Mater.* **2013**, *25*, 732.

[15] S. Ganguli, A. K. Roy, D. P. Anderson, *Carbon* **2008**, *46,* 806.

[16] K. M. Shahil, A. A. Balandin, *Nano. Lett.* **2012***, 12*, 861.

[17] H. Jung, S. Yu, N. S. Bae, S. M. Cho, R. H. Kim, S. H. Cho, I. Hwang, B. Jeong, J. S. Ryu, J. Hwang, S. M. Hong, C. M. Koo, C. Park, *ACS Appl. Mater. Interfaces* **2015**, *7*, 15256.

[18] M. Qin, Y. Xu, R. Cao, W. Feng, L. Chen, *Adv. Funct. Mater.* **2018**, *28*.

[19] F. An, X. Li, P. Min, P. Liu, Z. G. Jiang, Z. Z. Yu, *ACS Appl. Mater. Interfaces* **2018**, *10*, 17383.

[20] Y. Li, W. Wei, Y. Wang, N. Kadhim, Y. Mei, Z. Zhou, *J. Mater. Chem. C* **2019**, *7*, 11783.

[21] G. Lian, C. C. Tuan, L. Li, S. Jiao, Q. Wang, K. S. Moon, D. Cui, C. P. Wong, *Chem. Mater.* **2016**, *28*, 6096.

[22] Y. H. Zhao, Z. K. Wu, S. L. Bai, *Compos. Part A: Appl. S.* **2015**, *72*, 200.

[23] X. Shen, Z. Wang, Y. Wu, X. Liu, Y.-B. He, Q. Zheng, Q. H. Yang, F. Kang, J.-K. Kim, *Mater. Horiz.* **2018**, *5*, 275.

[24] Z. Wu, C. Xu, C. Ma, Z. Liu, H. M. Cheng, W. Ren, *Adv. Mater.* **2019**, *31*, e1900199.

[25] H. Fang, H. Guo, Y. Hu, Y. Ren, P.-C. Hsu, S.-L. Bai, *Compos. Sci. Technol.* **2020**, *188*.

[26] H. Fang, Y. Zhao, Y. Zhang, Y. Ren, S. L. Bai, *ACS Appl. Mater. Interfaces* **2017**, *9*, 26447.

[27] Q. Yan, F. E. Alam, J. Gao, W. Dai, X. Tan, L. Lv, J. Wang, H. Zhang, D. Chen, K. Nishimura, L. Wang, J. Yu, J. Lu, R. Sun, R. Xiang, S. Maruyama, H. Zhang, S. Wu, N. Jiang, C. T. Lin, *Adv. Funct. Mater.* **2021**, *31*. 2104062.
